# Assessing personal protective equipment usage and its correlation with knowledge, attitudes, performance, and safety culture among workers in small and medium-sized enterprises

**DOI:** 10.1186/s12889-024-19517-3

**Published:** 2024-07-25

**Authors:** Amir Hossein Khoshakhlagh, Mahdi Malakoutikhah, JeeWoong Park, Mohammad Dehghani Kodnoueieh, Zakiyeh Rafieian Boroujeni, Maryam Bahrami, Fatemeh Ramezani

**Affiliations:** 1 Department of Occupational Health Engineering, School of Health, Kashan University of Medical Sciences, Kashan, Iran; 2 Occupational Health and Safety Engineering, Social Determinants of Health (SDH) Research Center, Kashan University of Medical Sciences, Kashan, Iran; 3 Department of Civil and Environmental Engineering and Construction, University of Nevada, Las Vegas, USA; 4 Student Research Committee, Kashan University of Medical Sciences, Kashan, Iran

**Keywords:** Small and medium sized enterprises, PPE, Safety culture, Safety knowledge, SMEs

## Abstract

**Background:**

The use of personal protective equipment (PPE) should be a culture of a workplace, and deeply rooted in worker behavior and attitude during their practice. According to the recent studies only 64% of the workers use PPE properly. The present study aims to investigate the utilization of PPE among workers in small and medium-sized enterprises (SMEs), and its relationship with knowledge, attitude, performance, and safety culture among workers.

**Methods:**

This cross-sectional study was carried out using a questionnaire tool across SMEs in Kashan city in year 2023. The used tool included three questionnaires: demographic, safety culture, and knowledge, attitude and performance. Study papulation was 529 SMEs. Totally, the sample size was 369 persons and questionnaires were distributed among the workers of SMEs. Finally, SPSS software was used for statistical analysis and structural equation modeling. Various statistical tests including T-Test, ANOVA, RMSEA, CFI, TLI, and the chi-square ratio were employed.

**Results:**

The mean values (standard deviation) of age and work experience were 35.19 (12.33), and 15.60 (1.69) years, respectively. Among the 369 participants, 267 participants (72.4%) indicated that they use some PPE, although not all types. However, 102 individuals (27.7%) do not employ any PPE. The lowest score for safety culture dimension was attributed to safety training at 1.58. The results of the final model indicate that the assumed relationships between variables, as outlined in the study objectives, were well established, with all connections proving statistically significant.

**Conclusion:**

It can be concluded that the missing of inadequate legal supervision for small industries exists. Therefore, it can be inferred that if supervision and regulation are enhanced for safety training and implementation that may lead to increased usage of PPE.

## Background

According to the International Labor Organization (ILO) [1], the significance of small and medium-sized enterprises (SMEs) in global employment is paramount, attributing over two-thirds of jobs worldwide. In both industrialized and developing countries, SMEs have gradually taken more important roles and essential contribution to economic growth. A notable example is underscored by studies from ILO that approximately 70% of industrial innovations originate from entrepreneurs within small-scale units. Economic growth in many Asian countries, such as Korea, Taiwan, China and Japan, is directly proportional to SMEs, which constitute 99% of all business investments. These countries produce about 60% of the total industrial production and almost 40% of the total profits and taxes obtained by various industries in China [2, 3].

Despite the importance and pivotal roles of SMEs, defining and classifying SMEs lacks universal consensus. The differences are observed across various countries. For example, the ILO, South Korea and Japan consider companies with less than 50 workers as small and those with more than 50 workers as medium [4]. The European Commission divided companies into three categories: micro (fewer than 10 workers), small (fewer than 50 workers), and medium (fewer than 250 workers) [5]. In New Zealand, companies with less than 19 people, in Britain and Germany less than 250 people and in Canada less than 500 people are also classified as SME. The Statistics Center of Iran categorizes companies with less than nine workers as micro enterprises, those with the number of workers between 10 and 49 as small enterprises and those with the number of workers between 50 and 99 as medium enterprises [6, 7].

Regardless of how SMEs are considered across countries, their impact on economic and industrial development is critical. These enterprises offer numerous advantages over their larger counterparts, including enhanced innovation, job creation, and greater flexibility [8]. According to the report of the Organization for Economic Co-operation and development (OECD) [9], SMEs contribute approximately 90% to both employment and development efforts. As another evidence for the same, according to the data of the Bureau of Statistics, over 90% of the industries in Philippine, Canada, and China are considered SMEs. Iran’s reliance on SMEs is pronounced, with over 14 million individuals employed across more than 5 million SMEs [10].

However, despite their contributions, SMEs often grapple with inadequate occupational health and safety conditions for many reasons [6, 10]. Factors such as young, low-educated or illiterate workers, and a lack of or insufficient training about the occupation and its hazards, lack of strict regulations, also, the use of non-standard tools, poor lighting, high noise levels, lack of ventilation, and inappropriate personal protective equipment (PPE). These conditions are exacerbated by working conditions often with limited spaces. Studies reveal alarming statistics highlighting this problem evidenced across various nations. Japanese national studies showed that 72% of occupational injuries leading to prolonged absences in small companies and higher mortality rates compared to national averages [11]. In Europe, 82% of all occupational injuries and 90% of all fatal accidents occur in these industries [6]. Another study by Koh et al. support this problem by showing a higher occupational mortality rate in small-scale companies than national averages [12]. These findings collectively suggest that working environments within SMEs pose greater risks compared to those in large-scale companies. Despite high injuries, fatalities and their subsequent impact, this study showed that only 23.9% of workers in small scale companies are trained, and only 12% of employers provide necessary PPE for workers. Accordingly, a significant challenge lies in the inadequate utilization of PPE, leading to a considerable portion of workplace accidents. Reasons for this shortfall include insufficient training, improper distribution of PPE, and a lack of supervision. Research underscores the potential of PPE in reducing workplace injuries, with studies indicating that more than 90% of incidents can be mitigated through its proper implementation of PPE [13].

Despite the importance of PPE, the number of accidents leading to injuries and fatalities associated with improper or lack of use of PPE is significant. According to a recent study by the American Occupational Safety and Health Administration [14], only 64% of the workers use PPE properly. Unfortunately, failure to use PPE is one of the main factors leading to accidents [15]. According to the studies 34% of occupational accidents were caused by not using PPE at the workplace at the time of the accident. In addition, 13% of work-related accidents are caused by inappropriate use of these devices [16].

Establishing PPE management programs require the initial financial and temporal investment; however, the costs associated with establishing PPE management programs pale in comparison to the direct and indirect expenses incurred in the aftermath of accidents. The use of PPE should be a culture of a workplace, and deeply rooted in worker behavior and attitude during their practice. This view of the authors is supported by a study which revealed that the overwhelming majority of accidents stem from unsafe practices, attitudes, behaviors, and organizational culture [17].

Recognizing the pressing need to address these challenges, the present study aims to investigate the utilization of PPE among workers in SMEs, exploring its relationship with knowledge, attitude, performance, and safety culture among workers within SMEs. This work is particularly carried out with respect to the causes of non-use of PPE and associated factors.

## Materials and methods

This research used a cross-sectional investigation using a questionnaire tool across SMEs in Kashan city in year 2023. Kashan city is home to 529 SMEs, comprising 482 small-scale and 47 industries are medium-scale industries. The used tool included three questionnaires: demographic, safety culture, and knowledge, attitude and performance. The data of the present study were collected face-to-face and by distributing questionnaires.

### Demographic questionnaire

The demographic questionnaire included inquiries regarding age, education level, work experience, industry type, accident history, safety training, and use of PPE.

### Safety culture questionnaire

The safety culture questionnaire designed by Toori et al. is adopted in this study. The questionnaire examines safety culture in 10 dimensions: management system, information and communication exchange, leadership, safety training, safety rules and regulations, individual factors, knowledge, motivation, organizational factors, and equipment. It consists of 37 questions rated on a 5-point Likert scale, ranging from “completely agree” to “completely disagree” with three questions featuring reversing scoring. The questionnaire has a Cronbach’s alpha value of 0.855, and its content validity has been confirmed by experts [18].

### Knowledge, attitude and performance questionnaire

This knowledge, attitude, and performance questionnaire used in this study was developed by Voshoghi et al. [19]. The questionnaire consists of 25 questions, which were verified by the CUR and CUI methods. To assess the reliability of the tool, the Cronbach’s alpha coefficient and retest methods were employed. Awareness, attitude, risk perception, and performance were obtained as 0.884, 0.719, 0.832, and 0.727 respectively.

Regarding attitude assessment, questions were categorized into six main groups, covering management commitment, information, instructions and training, presence of a personal protection program within the organization, supportive environment, and treatment and involvement in the selection of PPE.

Regarding performance assessment, the questions were grouped into three, focusing on the use of PPE, reasons for non-compliance with PPE usage, and accident records.

For questions related to knowledge, responders were provided with three options (true/do not know/false). Attitude and risk perception were evaluated using a five-point Likert scale from “completely agree” to “completely disagree.” Performance-related questions were assessed using three options (never/sometimes/always).

### Sample size

The total workforce in SMEs in Kashan city amounts to approximately 9,572 individuals at the time of data collection in year 2023. These workers are distributed across various industries that are with fewer than 99 workers. These industries are categorized as follows: small-scale industries comprise those with 1 to 49 workers, while medium-scale industries include those with 50 to 99 employees.

The sample size of the current study was selected using Cochran’s method and based on previous studies, in which N equals 10,000, p equals 0.5 and q equals 0.5, error percentage or d equals 0.05 and z equals 1.96, was considered. Finally, the sample size of the present study is 369 people. Also, the type of sampling was simple random, which was used because of the wide range of workers in these industries. It was also tried to establish a balance between small and medium industries in terms of the number of participants.

For this study, following approval of the code of ethics from Kashan University of Medical Sciences, 369 questionnaires were distributed among the workers of SMEs in Kashan city. The sample size was determined based on calculations and employed simple random sampling methodology across the SMEs within Kashan city. The studied industries included small industries such as metalworking, cabinet making, car repair shop, carpentry, shoemaking, and battery making, and medium industries included carpet weaving and food industries.

### Inclusion and exclusion criteria

The inclusion criteria for this study include individuals with at least one year of work experience within the same SMEs, a willingness to cooperate and complete the questionnaire, a history of experiencing an occupational accident within the past five years.

The exclusion criteria include incomplete completion of the questionnaire, lack of satisfaction and cooperation from the participant, or deviation from the average characteristics of individuals within SMEs.

### Statistical analysis

After data collection, the obtained information was inputted into SPSS software for analysis. All analysis was performed using version 22 of the software. Various statistical tests including T-Test and ANOVA were employed to compare the key variables from the questionnaires, with a significance level set at 0.05 for all tests. Structural equation modeling was used to determine the relationships and causal network between both hidden and visible components.

In this research, three types of fit indices, such as absolute, comparative and parsimonious, were examined. The root means square residual measurement error (RMSEA) served as a measure of residual error, interpreted in relation to covariance value. Additionally, this study assessed the comparative fit index (CFI), Tucker-Lewis’s index (TLI), and the chi-square ratio to the degree of freedom (χ2/df) in order to evaluate the appropriate fit of the model. For a model to be considered a good fit, the RMSEA index value should be less than 0.08, and the χ2/df value should be less than 3. Moreover, higher values of CFI and TLI, closer to 1, indicate a better fit of the model.

## Results

The first demographic analysis indicates that the mean values (standard deviation) of age and work experience were 35.19 (12.33) and 15.60 (1.69) years, respectively. The distribution of industries between small and medium industries was 47.5% and 52.5%. Within the small industries, mechanics and car repairers accounted for 54% of the distribution at highest, while the carpet production industry occupied 56% within the medium industries. Table 1 presents further details about the demographic variables of the participants.

**Table 1:** Demographic variables of the study participants

Variables		Frequency (%)
Gender	Male	346 (93.76)
	Female	23 (6.23)
	Total	369 (100)
Education	High school	132 (35.77)
	Diploma	166 (44.98)
	Bachelor	38 (10.29)
	Master and more	33 (8.94)
	Total	369 (100)
Marital status	Married	245 (66.39)
	Single	124 (33.60)
	Total	369 (100)
Accident experience	Yes	173 (46.77)
	No	196 (53.11)
	Total	369 (100)
Safety training	Yes	178 (48.24)
	No	191 (51.76)
	Total	369 (100)

### Results of PPE usages

**Fig. 1:**
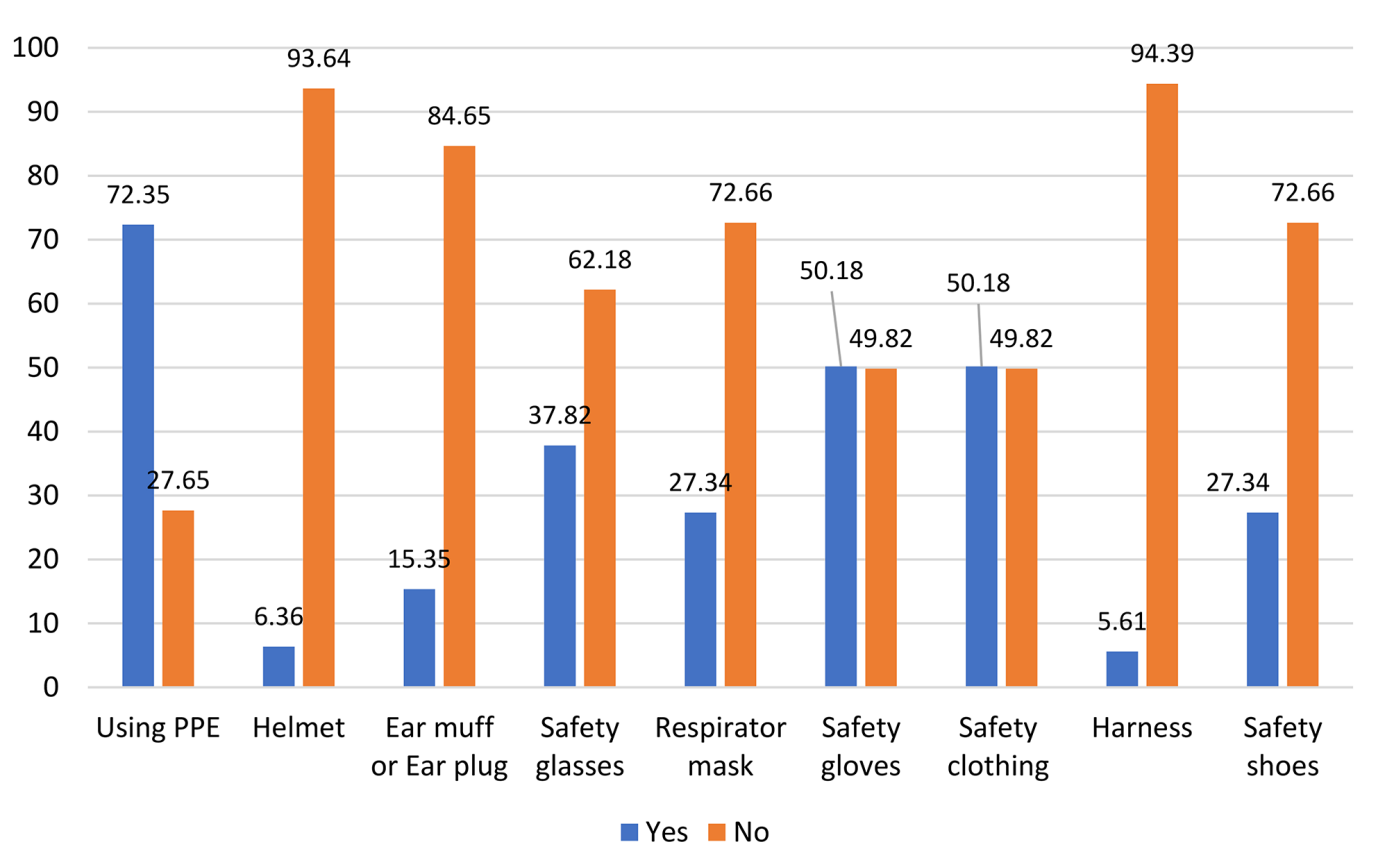
The usage and type of personal protective equipment used among the surveyed workers (%)

Figure 1 illustrates the usage and types of PPE used among the surveyed workers. Among the 369 participants, 267 participants (72.4%) indicated that they use some PPE, although not all types. However, 102 individuals (27.7%) do not employ any PPE. Among those 267 responders who indicated that they use some PPE, 249 individuals (93.26%) do not utilize or rarely utilize helmets for their own protections.

It is important to note that the first bar chart in Fig. 1 about ‘Using PPE’ is based on the entire surveyed population (369 responders) while the rest of the individual PPE items (e.g., helmet, ear muff, etc.) is based on those 267 responders.

More details of the responses about the use of PPE are presented in Table 2, which provide a summary of responses in the form of a 5-point Likert scale. Although these 267 responders indicated that they use some PPE while working on site, the majority of the variables (i.e., helmet, ear muff or ear plug, safety glasses, respirator mask, harness, and safety shoes) have never been utilized by more than 50% of the responders. The most actively used items are ‘safety gloves’ and ‘safety clothing,’ which still have a low rate of usage: about 32% and 53%, respectively, when accounting for the ‘often’ and ‘always’ frequencies.

**Table 2:** The rate of use of each personal protective equipment among the participants

Variables	Frequency (%)					Total
	Never	Rarely	Sometimes	Often	Always	
Helmet	226 (83.89)	25 (9.36)	15 (5.62)	1 (0.37)	0	267
Ear muff or ear plug	199 (74.53)	14 (5.24)	27 (10.11)	17 (6.36)	10 (3.74)	267
Safety glasses	147 (55.06)	22 (8.24)	52 (19.47)	33 (12.36)	13 (4.87)	267
Respirator mask	156 (58.42)	22 (8.24)	44 (16.48)	30 (11.23)	15 (5.62)	267
Safety gloves	115 (43.07)	25 (9.36)	41 (15.35)	49 (18.35)	37 (13.85)	267
Safety clothing	101 (37.82)	6 (2.24)	19 (7.11)	68 (25.64)	73 (27.34)	267
Harness	237 (88.76)	22 (8.24)	3 (1.12)	3 (1.12)	2 (0.75)	267
Safety shoes	164 (61.42)	30 (11.33)	11 (4.12)	19 (7.11)	43 (16.10)	267

### Results of related factors to PPE usages

The average score of the administered questionnaires reveals that within the safety culture section, the final score fell within the moderate range, based on a 5-point Likert scale. Meanwhile, in the knowledge, attitude, and performance section, despite comparably lower scores in knowledge and attitude, the overall score of the questionnaire remained within the moderate range, based on the combined Likert scales explained earlier (Table 3).

**Table 3:** Questionnaire score and its relationship with the use of personal protective equipment among the studied workers

Variables		Mean (SD)	*P* value
Safety culture	Management system	1.93 (1.08)	< 0.001*
	Exchange of information and communication	2.15 (0.70)	0.035*
	Leadership	2.41 (0.71)	< 0.001*
	Safety training	1.58 (0.66)	< 0.001*
	Safety rules and regulations	2.79 (0.87)	< 0.001*
	Individual factors	3.52 (0.72)	0.065
	Safety knowledge	1.84 (0.62)	0.054
	Motivation	2.14 (0.93)	< 0.001*
	Organizational factors	2.07 (0.75)	0.027*
	Equipment	3.58 (1.10)	0.031*
	Total score	2.44 (0.51)	< 0.001*
Knowledge, attitude and performance	Knowledge	14.57 (5.83)	0.078
	Attitude	18.36 (6.66)	< 0.001*
	Performance	21.11 (4.41)	0.084
	Total score	50.22 (9.85)	0.036*

* *P* < 0.05

It is important to note that within the safety culture dimension, the lowest score was attributed to safety training at 1.58, while the highest score was associated with individual factors. In the knowledge, attitude, and performance section, the highest score was observed in performance at 21.11.

Furthermore, with the exception of individual factors, safety knowledge within the safety culture questionnaire, and knowledge and performance within the knowledge, attitude, and performance questionnaire, all other variables exhibited a significant correlation with the utilization of PPE.

### Results of structural equation modeling

**Fig. 2:**
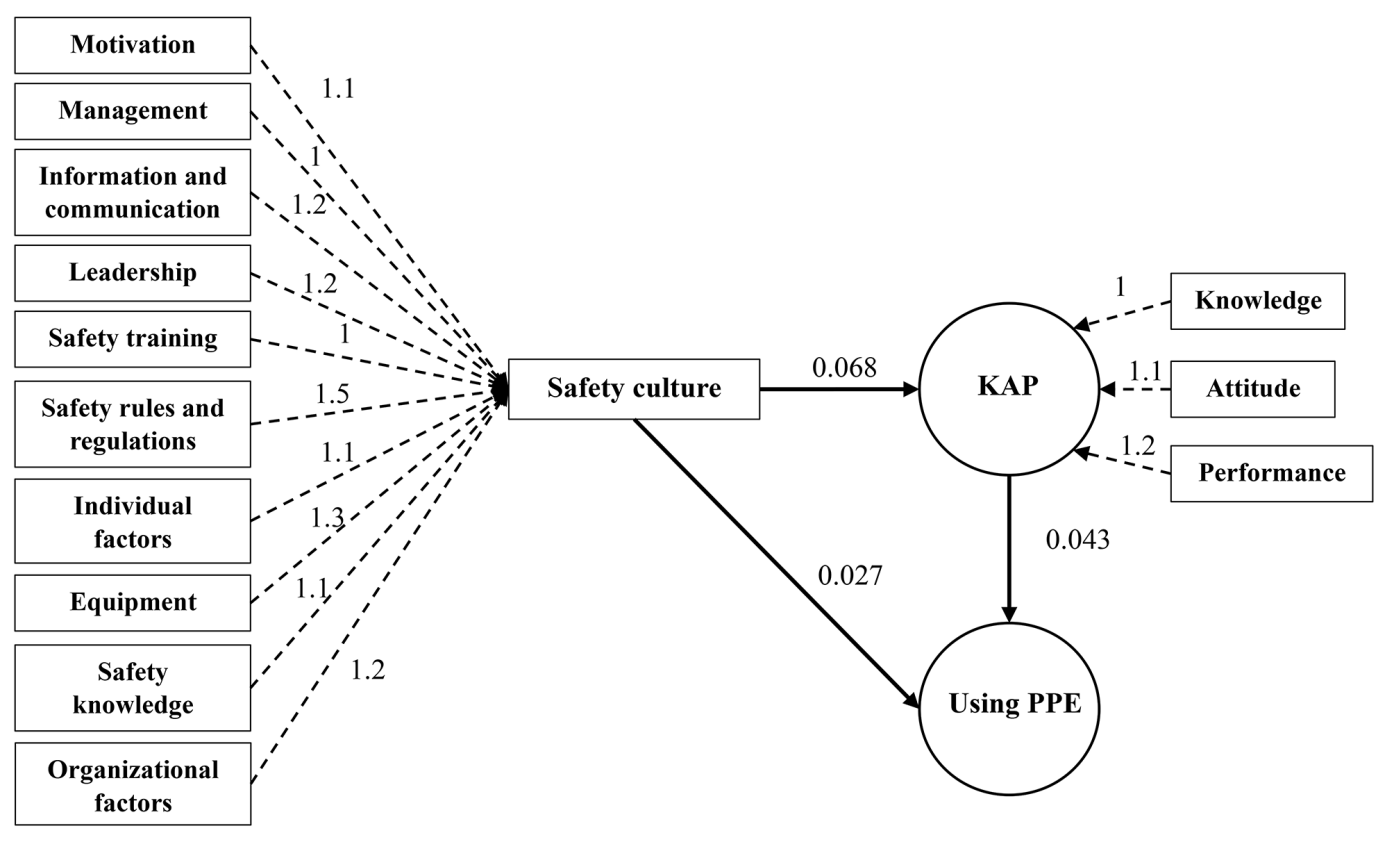
The final model and related rout between the main variables KAP: Knowledge, Attitude, Performance; PPE: Personal Protective Equipment

The results of the final model indicate that the assumed relationships between variables, as outlined in the study objectives, were well established, with all connections proving statistically significant (Fig. 2). The results of the root mean square residual measurement error (RMSEA), comparative fit (CFI), Tucker-Lewis’s index (TLI) and the chi-square ratio to the degrees of freedom (χ2/df) demonstrate that all indices fell within acceptable ranges, indicating a satisfactory model fit (Table 4). The values of CFI and TLI indices were close to 1, and the RMSEA index was less than 0.08. Therefore, our findings highlight a significant correlation between safety culture and people’s knowledge, attitude and performance. Moreover, these factors also have a significant effect on the usage of PPE.

**Table 4:** The results of fit indices of the designed model

Indicators	CFI	TLI	RMSEA	SRMR	Chi^2^	Confidence interval (CI)
Estimated value	0.927	0.900	0.101	0.049	153.827	(0.084–0.118)

## Discussion

The present study aimed to explore the utilization of PPE and its correlation with knowledge, attitude, performance, and safety culture among workers in SMEs. A total of 369 participants were involved in the study, revealing that 72.35% of them utilized at least one type of PPE. Moreover, their scores in safety culture and knowledge, attitude, and performance were found to be moderate, with a significant association between these variables and the rate of PPE usage.

### PPE usages

According to the Organization for OECD report, SMEs contribute to approximately 90% of employment [20]. Similarly, ILO report highlights that SMEs constitute the majority of the workforce (40% in developed countries and more than 60% in developing countries) [21]. Despite their significant role, OHS conditions in SMEs often remain subpar due to various factors, including a predominantly young, undereducated, or illiterate workforce and inadequate training regarding job hazards [22]. In this regard, the present study also indicated that a majority of participants who were young with knowledge levels up to the diploma level had limited safety knowledge and culture, suggesting that their age and limited experience among workers could contribute to reduced safety knowledge and culture.

The present study also identified a significant relationship between the management system and the use of PPE, indicating the weakness of the safety culture in these industries. Furthermore, the results support past findings about the relation between the non-use of PPE and a lack of safety culture and insufficient safety knowledge.

### Related factors to PPE usages

Additionally, in medium-sized industries with over 25 workers, the mandatory requirement for a health and safety officer, as stipulated by Iran’s labor administration law, alongside rigorous oversight from relevant organizations and labor laws, has contributed to elevated levels of occupational health and safety management [23].

Dasandara et al.‘s research in Sri Lanka was about limiting reasons for use of personal protective equipment among construction workers. The results indicated that three main categories, namely, ‘individual’, ‘organizational’, and ‘environmental’. The analysis of these reasons generated knowledge, which can be used by respective industrial safety practitioners in Sri Lanka in understanding the current situation and as a way forward for enhancing the use of PPE among construction workers [24]. This stringent regulatory environment likely accounts for the higher average utilization of PPE observed in this study.

Furthermore, the predominant usage of safety gloves and work clothes aligns with the uniformity of working conditions in many SMEs, where consistent attire is utilized due to the homogeneous nature of the work environment. Additionally, individuals prioritize personal hygiene, leading them to utilize gloves to prevent soiling their hands during work activities [25, 26].

Moreover, the limited usage of certain PPE such as helmets and safety belts in smaller businesses can be attributed to the confined nature of their work environments, which often involve ground-level tasks without significant height-related risks. Consequently, the study’s findings indicate a lower frequency of usage for these particular items based on the work nature. In other situations, where exist excessive noise, inappropriate lighting, the presence of heavy objects, metal fumes, and hazardous chemical factors [27], the utilization of protective glasses, breathing masks, and earphones remain low. Chen et al.’s research in China showed that there are limited usage of certain PPE in SME [28].

This deficiency underscores a weakness in PPE utilization within these industries, possibly stemming from inadequate knowledge, attitudes, and training, particularly among smaller enterprises lacking a designated occupational health and safety officer [29]. These are supported by our data, presenting that the average score of safety knowledge, motivation from the safety culture and knowledge questionnaire and attitude from the knowledge, attitude and performance questionnaire is lower than the average and is at an unfavorable level. These results highlight the need for enhanced educational and training initiatives, particularly within smaller establishments with fewer than 25 employees and lacking dedicated occupational health and safety personnel.

Additionally, the model of the present study demonstrates that safety culture, knowledge, and attitude significantly impact the utilization of PPE—a finding supported by previous research. Note that Smith et al. conducted a study using structural equation modeling, revealing a positive relationship between safety culture and firefighter safety motivation, including the motivation to use PPE [30]. Similarly, Leiss found that among nurses, an increase in safety culture and climate correlated with an increase in the utilization of PPE [31].

Moreover, Fu et al.‘s research in China further supports these findings [32]. Their study involved training 525 welding workers from 25 SMEs across various industries, focusing on interactive learning, workplace assessment, and group discussions on safety rules and regulations, machine operation, fall prevention, fire/explosion prevention, ergonomics, and environmental hazard recognition and prevention. The pre- and post-training questionnaire results showed significant improvements in awareness, attitude, and practice scores, as well as enhancements in health management and the provision and use of PPE. These findings parallel the outcomes of the present study. We note that one of the shortcomings in small industries is the absence of a designated individual responsible for occupational health and safety, coupled with deficiencies in legislative and regulatory oversight, which impact educational enhancement in these settings.

In general, the results of this study showed that the amount of use of PPE among workers in these industries was low, and one of the reasons for this was the lack of awareness and attitude of these people to use PPE. Therefore, it can be concluded that the use of PPE in SMEs can be increased with better training and supervision by legal authorities. Of course, this conclusion requires more studies.

## Conclusion

The present study indicates that the utilization of PPE in the studied industries is low, alongside a low level of safety culture and knowledge among individual workers. One reason for this phenomenon could be attributed to the missing of inadequate legal supervision for small industries. Therefore, it can be inferred that if enhanced supervision and regulation for safety training and implementation may lead to increased usage of PPE. Additionally, it can be deduced that occupational health and safety regulations in medium-sized companies have resulted in enhanced occupational health and safety practices compared to those in small companies. Consequently, we suggest expanding labor laws to encompass occupational health and safety provisions specifically tailored for small enterprises. Due to the lack of study in the field of measurement of occupational harmful factors in these industries, future studies can help the results of the present study by performing measurements. It is also possible to carry out an educational intervention and compare its results for the effect of education on the use of PPE with this study. Also, according to the results of the present study, health centers and labor law can increase the supervision of SMEs and increase the level of knowledge and attitude of workers by providing the necessary training.

## Data Availability

The datasets used and/or analysed during the current study are available from the corresponding author on reasonable request.

## Abbreviations

ILO: International Labor Organization
OECD: Economic Co-operation and development
PPE: Personal Protective Equipment
RMSEA: Root Means Square Residual Measurement Error
SMEs: Small and Medium-sized Enterprises
SPSS: Statistical Package for the Social Sciences
